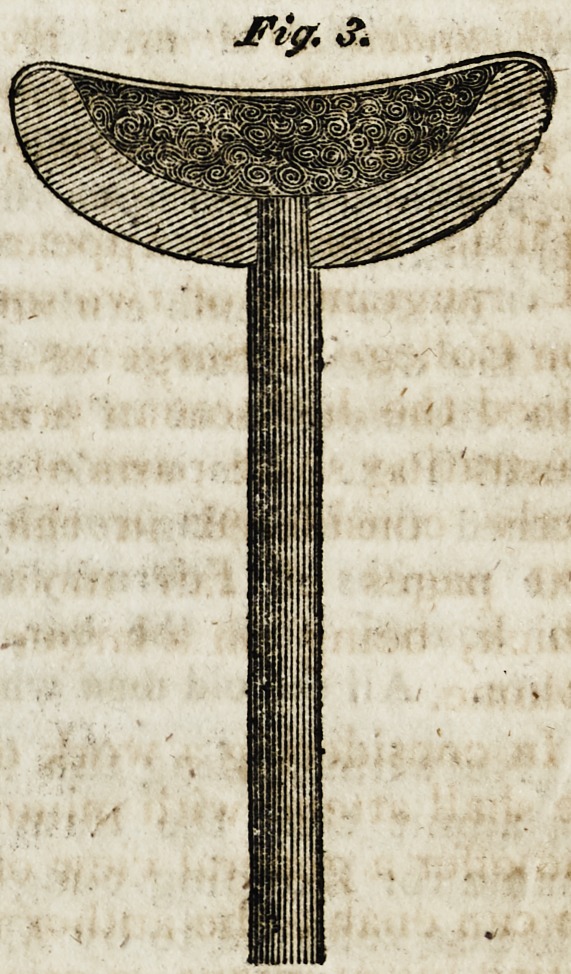# Collectanea Medica, Consisting of Anecdotes, Facts, Extracts, Illustrations, Queries, Suggestions, &c.

**Published:** 1815-08

**Authors:** 


### COLLECTANEA MEDICA,

CONSISTING OF

### ANECDOTES, FACTS, EXTRACTS, ILLUSTRATIONS, QUERIES, SUGGESTIONS, &c.

RELATING TO THE

*History or the Art of Medicine, and the Auxiliary Sciences.*

---

### *On the Means of producing a double Distillation by the same Heat;* by Smithson Tennant, Esq. F.R.S.

IT was first made known by the experiments of Dr. Black, and has since been confirmed by those of Mr. Watt and other philosophers, that the quantity of heat required for raising the temperature of water from fifty degrees to that of the boiling point, is only about a sixth of that which is afterwards required for converting the boiling water into steam. As the steam itself is not hotter than the boiling water, the heat which it had absorbed was said by Dr. Black to be latent; being merely employed in supporting the aerial state which the water had assumed. Whenever this steam is condensed, the heat which was latent again re-appears, and in such considerable quantity, that it has been found convenient for various purposes to employ the condensation of steam for heating other bodies.

But, though water may, by this means, be brought to the boiling point, it cannot be raised above it, and therefore cannot be converted into vapour, so as to pass over by distillation. As soon as the the steam has imparted to the water its own temperature, there is no longer any transfer of heat, and the steam passes through the water uncondensed.

If, on the contrary, the steam did continue to condense, then the water would itself be converted into steam, and might, by that means, be distilled over without any additional fire; and, though this does not take place under the usual circumstances, yet it may be effected in the following manner.

The temperature required for converting any fluid into vapour is dependant on the pressure of the air upon its surface, and may, therefore, be lowered, if that pressure is diminished. If then, the weight of the air was removed from water, it would rise into vapour below the common boiling point, and might therefore be distilled over by steam of the usual heat.

In order to produce this effect, a vessel, having a receiver connected with it, should be made air-tight, and the steam made to pass through the vessel along a worm or spiral tube of metal, in the manner represented in the annexed outline.

The vacuum is now easily produced, by applying heat to the vessel till the steam issues from the opening in it, and in the receiver, when they are to be immediately closed, and the heat removed.

The water distilled over is collected in the receiver, which is kept cool for that purpose.

An apparatus of this kind I had constructed chiefly for the purpose of explaining the theory of latent heat, or of the capacity of bodies for heat in different states; but it is possible, that it may also be of some further practical utility, whenever it is of consequence to economise the consumption of fuel. When water is deficient on board of ship, it has been, in some degree, supplied by distillation from the ship's boiler, and if the steam from the boiler had been made to pass through the apparatus just described, the quantity would be nearly doubled.

By an experiment which I had made some time ago, about three-fourths of the quantity obtained by the first distillation, were added by the second. But, I believe, a larger proportion may be procured, when the second distilling vessel is duly coated with flannel, or some light substance, to retain the heat.

Though salt water does not boil at so low a degree of heat as fresh water, yet upon trial with sea water, the difference was found to be quite insignificant, compared with that of the steam formed under the usual pressure, and *in vacuo;* and did not sensibly affect the result of the process. The only doubt as to the propriety of taking such a vessel to sea, would arise from the degree of danger which there is of experiencing a want of fresh water. This, probably, I apprehend, is not great; but, on the other hand, there is the important object of saving the lives of the people in the ship, whenever such deficiency is experienced.—*Phil. Trans.*

The melancholy loss of the late Mr. Smithson Tennant renders every fragment which he has left, if possible, more valuable than before. His foregoing paper, in the last number of the Philosophical Transactions, appears to us important in many economical purposes, which the modesty of the author has overlooked. He was, perhaps, not aware that the distillation of salt water used for culinary purposes on ship-board has been principally discouraged on account of the increased saltness of the residuum, which added to the distaste arising from the continual use of salt provisions. By Mr. T.'s plan, this objection may be removed, as the distilled portion from the boiler containing the provisions may be returned, and its contents even further diluted by a portion from the condensed steam of a second boiler. Besides this, however plentiful water may seem on-board his Majesty's ships, there is always a want of it for washing, and for other purposes, more conveniently accomplished by fresh than by sea water. There are even circumstances under which salt itself is wanted, which the residue of the second boiler might easily supply. In pharmacy the heat may be economized with great advantage. The first boiler might distil simple waters, and the second spirits, without the possibility of empyreuma or boiling over.

We shall present our readers with a biographical account of Smithson Tenant, Esq. in our next.

---

*Case of Popliteal Aneurism, in which a particular Mode of obliterating the Femoral Artery was successfully employed.* (From Mr. Hodgson's Treatise on Arteries.)

The following case, in which a new method of obliterating arteries for the cure of aneurisms was successfully adopted, is related by Professor Assalini of Milan.* The instrument employed for compressing the artery resembles the pincers of M. Percy, which I have mentioned in a former part of this treatise.†

---

* See Manuale di Chirurgia, p. 81. Milan, 1812.
† Page 208.

It consists of two short silver blades, connected together by a rivet, like a pair of common dressing forceps. The ends of the blades between which the artery was compressed, are broad and flattened. A spring, composed of a piece of elastic steel, is attached to the other end of one of the blades, and, by pressing against the opposite blade, retains the flat extremities of the instrument in contact. The degree of pressure is regulated by a screw, which passes through the handles of the instrument.

A man, fifty-one years of age, was afflicted with a popliteal aneurism, which, on the 2d of November, 1811, had arrived at considerable size. After compression had been tried for several days, the operation was performed by dividing the integuments covering the femoral vessels on the inside of the sartorious muscle. The femoral artery was fairly exposed, and placed between the blades of the compressor, without being raised from its bed. The action of the spring of the compressor, without employing the screw, was sufficient to stop the pulsation in the aneurism, but the jet of blood in the upper portion of the artery communicated to the instrument a very sensible oscillation. The sides of the wound were placed as near to each other as the intervention of the instrument, which was surrounded with lint, would permit. The patient was tranquil during the day and following night. Thirty-six hours after the operation a slight degree of fever had commenced. The pulse was frequent and hard, but the skin was moist, and the leg was in its natural state. At this time a very slight pulsation existed in the tumour. As it now appeared, that the spring of the instrument was not of itself sufficient to resist the impulse of the circulating blood, and retain the sides of the artery in contact, the compression was increased by means of the screw which passed through the handles of the instrument. In this manner the circulation through the artery was totally intercepted. The patient did not complain of pain, or any inconvenience, except a sensation of cold at the ankle. Twenty-four hours after the instrument was tightened, no pulsation could be felt in the tumour, which had become soft, and was diminished one-third in size. Sixty hours after the instrument was tightened, it was conceived that the sides of the artery had united, and that the presence of the instrument in the wound was useless, and might occasion dangerous consequences. It was, therefore, decided, first to unscrew, but not to remove the compressor, so that it might be tightened, if necessary. No pulsation, however, could be discovered in the aneurism when the compression upon the artery was omitted: the instrument was therefore withdrawn. Fourteen days after the operation the wound was healed, and in forty days the patient left the hospital, the tumour being reduced to the size of a small egg.

Professor Assalini informs me, that in two cases of popliteal aneurism in which he employed this mode of obliterating the femoral artery since the occurrence of the above case, the instrument was removed at the expiration of twenty-four hours after it had been been applied to the artery. The pulsation did not return in the aneurisms after the removal of the instrument, and the patients were cured in a very short time.

*Case of Axillary Aneurism, in which the Subclavian Artery was tied.* (From the same.)

I am indebted to Mr. Thomas Blizard for the following notes of a case of axillary aneurism, in which he recently tied the subclavian artery.*

The patient was a robust man, forty-seven years of age. When he was admitted into the London Hospital, on the 10th of January, 1815, a tumour as large as a small lemon was situated in the left axilla. It was surrounded with a diffused swelling, extending underneath the pectoral muscle upwards as high as the clavicle, and anteriorly almost to the sternum. The whole limb was swoln and œdematous. He complained not only of constant pain in the tumour and the surrounding parts, but also of occasional attacks of the most excruciating pain in the palm of the hand. The integuments covering the tumour were of a dark livid colour: there was an appearance of excoriation, and a slight oozing of serum from its most prominent part. The skin upon the upper and inner part of the arm, to a considerable extent, was of an erysipelatous red colour. A distinct pulsation was perceptible at the base of the tumour, but it became less evident towards its apex, at which part it was altogether imperceptible. No pulsation could be felt in the radial artery of the diseased arm, the sensation of which was very much diminished. The pulse in the right arm was so quick that it was difficult to count it. There was but little difference in the temperature of the two arms. The breathing was hurried, and peculiarly irregular. The countenance was expressive of great anxiety, and he complained of extreme sufferings in his arm. On the 21st of December, 1814, whilst unloading a waggon, and having a weight in his right hand, and his body supported by the other, he felt a sudden snap and an acute pain in his left axilla. Two days afterwards he observed a pulsating tumour in the axilla, the increase of which was attended with so much pain as to disorder his health, and render him unable to follow his employment as a labourer.

The subclavian artery was tied in the following manner, within half an hour after the patient was admitted into the hospital. An incision, about three inches in length, was made through the inte-

* Since the preceding sheets of this treatise were printed, Dr. Colles, of Dublin, has published an account of two cases of aneurism, in one of which he tied the subclavian artery on the tracheal side of the scaleni, and in the other on the acromial side of these muscles. Both cases terminated fatally. For a particular account of them I must refer the reader to Dr. Colles's paper in the 41st Number of the Edinburgh Medical and Surgical Journal.

guments at the root of the neck, on the acromial side, and parallel with the external jugular vein. The platisma myoides being divided, the cellular membrane was separated with the finger, until the pulsation of the subclavian artery was felt where the vessel passes over the first rib. The finger being pressed upon this part of the artery, the cellular sheath investing it was carefully opened with the point of a knife. A ligature was then conveyed underneath the artery by a common aneurism-needle with the greatest facility. The ligature being tied in the common manner with a double knot, the wound was closed with strips of adhesive plaster. The omo-hyoideus muscle was not seen, and scarcely any blood was lost during the operation, which did not occupy more than five minutes.

Immediately that the ligature was tied, the pulsation ceased in the tumour, which was also somewhat reduced in size. The swelling and tension of the surrounding parts diminished ; the pain was very much abated, and the erysipelatous appearance on the arm was much less distinct. The limb was wrapped in wool and flannel: sixty drops of tincture of opium were given to him. In the evening his pulse was 108, and strong: his skin was moist: the temperature of the left arm was somewhat diminished: the opiate was repeated. On the morning of the first day after that on which the operation was performed, the pain was still more diminished: his respiration was more calm, though slightly hurried: the skin was moist: the temperature of the limb above the elbow was natural; below the elbow and at the fingers it was much diminished : the diffused swelling below the clavicle continued to subside: no pulsation could be felt at the wrist: there was a slight oozing of a bloody fluid from the tumour: the pulse was 112, and strong. As there had been no evacuation from his bowels, a purgative medicine was given to him, and he appeared to be relieved by its operation. In the evening the opiate was repeated. On the second day he had more sensation in the arm, and felt a pricking in his fingers. The arm was as warm as the other to the touch, and perspired freely: the blood was observed to circulate naturally through its veins. The wound had adhered, and looked as well as possible. The swelling below the clavicle continued to decrease: a slight suppuration had commenced on the most prominent part of the aneurism. Pulse 104. In the evening he complained of pains in the hand and fingers, which he compared to the cramp. The ulceration upon the tumour was increased. Pulse 108. The opiate was repeated. He slept well, and was easy on the morning of the third day. The ulceration had extended, and a discharge of grumous bloody matter had taken place from the tumour, to which a resinous ointment was applied. The erysipelatous appearance on the arm was very much diminished, and the swelling below the clavicle continued to subside. His breathing was natural. Pulse 104. In the evening the discharge from the tumour was more copious: the cramp-like pains in the fingers continued. Fifty drops of tincture of opium were given to him. On the fourth day, when he awoke in the morning, he was much alarmed by a very considerable discharge of grumous blood from the tumour. He appeared much agitated, but when the part was dressed he became more composed. He complained of languor and general uneasiness. Pulse 102, and weaker. His breathing was rather more hurried. He was troubled with a slight cough, by which the pain in the shoulder was increased. His tongue was white. As his bowels had not been evacuated since the operation of the purgative medicine, it was repeated. In the evening the medicine had operated, and the symptoms were much alleviated. A poultice was applied to the tumour, which was in a sloughing condition. The opiate was repeated. He passed a quiet night; but, on the morning of the fifth day, he complained of pain in the chest, which was increased by a full inspiration or by coughing. The sloughs were separating freely from the tumour. The swelling and hardness of the arm were much diminished, but the power of feeling and of motion was not increased. Pulse 100, and irregular. In the evening, the pulse was 116: the skin warm and moist. The opiate was repeated. On the sixth day, the breathing was still short. Pulse 120. In the course of the day occasional twitchings of the muscles, particularly of the hand and face, were observed. At intervals there was a slight confusion of intellect. In the evening his breathing was hurried. Pulse 128, but regular. The wound and the tumour continued in the same state. A slight lividity of the fore-arm and fingers was observed, which disappeared upon pressure, but returned when the pressure was removed, except on the knuckles of the ring and middle fingers, where it was stationary. He was ordered some wine and porter: the opiate was repeated. He passed a very restless night, during which he was frequently delirious. On the morning of the seventh day, the delirium continued, and his speech was very imperfect. The twitchings of the muscles were more general. He complained of great oppression, and frequently appeared drowsy. His breathing was short. Pulse 136, but not very irregular. His tongue was much parched. The limb was in the same condition as on the preceding day. In the evening a copious discharge of sloughs had taken place from the axilla. The swelling of the arm and shoulder had entirely subsided. The delirium and twitching of the muscles continued. Pulse 128, and weak. His breathing was very short. The wound above the clavicle had a favourable appearance. The allowance of wine was increased. The poultice was continued, and the opiate was repeated at bed-time. During the night he was very delirious. In the morning of the eighth day his pulse was 140, weak and irregular. His breathing was much hurried, and his countenance had a wild appearance. His tongue was parched, and covered with brown fur. His speech was so rapid as to be scarcely intellible. The ring and middle fingers were black. The symptoms increased, and he lingered until night, when he died.

The following appearances were observed upon dissection. The

The wound above the clavicle contained a very small quantity of pus. The ligature was situated on the subclavian artery, about one-third of an inch on the acromial side of the scaleni muscles. The external surface of the artery in the vicinity of the ligature was imbedded in lymph. The extremity of the artery on the acromial side of the ligature contained a conical plug of coagulated blood about the third of an inch in length. This plug terminated at the origin of a considerable branch. The extremity of the artery on the tracheal side of the ligature did not contain a plug of coagulated blood, but was closed by a clot of lymph of a white colour. The ring of the ligature was situated in a depression caused by the division of the internal and middle coats of the artery. The pericardium exhibited the effects of a high degree of inflammation. Its cavity contained about an ounce of serum. The heart and the reflected portion of the pericardium were covered with flakes of lymph. The posterior surface of the heart, when the lymph was scraped off, was of a deep red colour. The internal surface of the ascending aorta was of a bright scarlet colour. Its internal coat was much diseased, and exhibited numerous white patches. The internal surface of the right carotid and the left subclavian arteries was of a light red colour. This appearance, in a less degree, was also observed in the abdominal aorta. In the left axilla there was a large cavity, the boundaries of which were in a state of sphacelation. The sphacelation involved the aneurismal sac and the lower extremity of the artery communicating with it, the brachial nerves, the veins, and adjacent parts. The upper extremity of the artery, and the commencement of the sac, were not destroyed. Both extremities of the artery, to a considerable distance, were filled with coagulated blood. Two of the fingers were completely mortified.

---

### *Extracts from Mr. Moore's History of the Small Pox.*

Dr. Woodville's History of Small Pox having so lately appeared, and containing the most important historical chain, we shall content ourselves with offering a few interesting anecdotes from Mr. Moore's publication. Though many of of these may not be entirely new, yet they are not extracted from medical works, and mark an extensive reading, which only those who reside in the metropolis can easily accomplish.

*On the Etymology of the Small Pox.*—"The Small Pox and Measles not having existed in the classical ages, there could be no term for it in the Greek or Latin languages. The Arabians invented words of their own. But, when these maladies appeared in Europe, the Latin language was universally in use among the learned. *Pestis*, *Pestilentia*, and *Lues*, were applied to these, in common with other epidemics; *pestilentia ignis*, the fire plague, was probably applied to erysipelas, and all dangerous

 eruptive eruptive diseases. But, as a word was wanted to designate the new disease, *Variola* was coined, evidently derived from the Latin word *Varius*, which signifies spotted, or from *Varus**, a pimple. Thence the Spaniards formed their name, *Viruelas*, which the Italians liquified into *Il Vignolo*, and the French framed their *Verole:* for the diminutive *petite* was not added till about the fifteenth century. The French had a word of their own, also, for Small Pox, *Piquote*, which is used by Rabelais and the old French writers. It appears, that when the malady extended to the North of Europe, that the Saxons, instead of adopting the Latin word *Variola*, invented the vernacular name *Poccadl*,† derived from *Pocca* or *Pochcka*, a bag or pouch. The Anglo-Saxons also adopted this word, which was variously spelt by different writers, and became at length Pock and Pox.

"The epithet Small in England and *petite* in France, were subsequent additions."

*On the Medicine of Monks.*—"Notwithstanding the veneration that such abodes were once held in, moderns are prone to believe, that even the convent of St. Gall was a useless and dull institution; and it must be owned, that these respectable annals do not enable us, in a convincing manner, to refute this heavy accusation. For, although father Ekkehard may have unravelled the intrigues of those ambitious monks who aspired to the dignity of abbot, most faithfully; yet, in the present times, they can only be read short. He has, however, described one of his friends with more success.

"Notkerus was both a monk and a physician, who, besides, knowing something of theology and medicine, was a rare scholar, an interesting painter, and a delightful poet. So various were his talents, that he relieved the sick monks, when languishing in their cells, with physic and prayers; he adorned the walls of the monastery with his pencil, he composed Latin hymns, and chanted them in the chapel, and made the roof of the refectory ring with his wit. His pictures and poems have been suffered to perish, and the few remaining specimens of his jests are obscured by Gothic Latin; but two examples of his medical abilities have been preserved.

"Henry the second, Duke of Bavaria, a person of some humour, consulted Notkerus upon his health; he gave a feigned account of his complaints, and shewed him a bottle, according to

---

* Celsus and Pliny take notice of Vari; and the latter author, for the benefit of the Roman ladies who were afflicted with pimples on their faces, gives the following receipt for a very delicate cosmetic, "Hen fat well beat up and mixed with onions cures pimples."

"Varos adeps Gallinaceus cum cæpa tritus et subactus (sanat." C. Plin. Hist. Nat. lib. xxx. c. 4.

† "Poc. pocc. *A pock.* Pustula, papula, tuber. Pocadl. "Morbilli, pustulæ, variolæ. Pocca. pochca. poha. *A pouch.* Pera." Diction. Gothico-Latin. Ed. Lye.

the the usage of these times; but it contained a deceptious liquid. The monastic doctor alternately examined the bottle and the patient, scientifically and shrewdly; at length, bursting with inspiration, he exclaimed, "Behold a miracle! an unparalleled miracle! a man, nay, this mighty duke, hath conceived, and in thirty days he shall bring forth a son, and suckle him at his breasts!" The detected Duke confessed his stratagem to the priest of God; and the prediction was mysteriously fulfilled, nearly at the time foretold, by a fair maid of honour. Some temporary disgrace was incurred; but, through the earnest intercession of Notkerus, the duke was appeased, and the lady, when recovered, was restored to favour at Court.

"Soon after this pretended consultation, he was sent for in good earnest by Kaminaldus, the bishop of the diocese, who had been suddenly taken ill. The physician, well aware of the prelate's plethoric regimen, instantly bled him most copiously. And, after viewing the rich inflamed blood, prognosticated, as was believed, from the smell, that in three days the Small Pox would break out.* The bishop, though fully prepared, was not the less alarmed; and besought the physician to stop that dangerous eruption. Notkerus replied, 'that I could easily do, but, if I obeyed, my regrets and misery would be insupportable; for to check the eruption would be equivalent to delivering up your reverence to death.' This answer was convincing, and stopt all argument, after conviction. The prelate did not persist. The Small Pox was allowed to proceed regularly, and the bishop was cured without being even pitted.

"The sagacity of this physician, in predicting the Small Pox, and his success in the treatment, are clear proofs that the faculty in Switzerland were in that age quite familiar with the disease; and we may deduce, from the geographical position of Italy and France, that the malady must have been known in these countries still earlier.

"After the contagion had overspread the continent of Europe, Great Britain could not long escape; which was invaded, in quick succession, by Saxons, Danes, and Normans.

"In the Harleian collection, in the British Museum, there is a very antient Anglo-Saxon manuscript, which, from internal evidence, is judged to have been written in the tenth century. It contains many pious exhortations, exorcisms and prayers, in the Saxon and Latin languages; and, among others, there is a supplication in Latin, which may be rendered thus.†

 "An

* Most probably because he knew that the disease was at that time epidemic.—Edit.

† "Exorcismus contra Variolas :—

"'In nomine Patris, et Filii, et Spiritus Sancti, amen. ✠ * in

* "The mark ✠ denotes where the exorcist made the sign of the Cross.

"An exorcism against the Small Pox.

"'In the name of the Father, of the Son, and of the Holy Ghost, amen. *N°* May our Saviour help us. *N°* O Lord of Heaven!.... hear the prayers of thy man servants, and of thy maid servants; O Lord Jesus Christ. I beseech thousands of angels that they may save and defend me from the fire and power of the Small Pox; *N°* and protect me from the danger of death; O Christ Jesus! incline your ears to us, &c.'

"This affecting prayer shews strongly the terror which the Small Pox had inspired.

"In the Cottonean Library there is a similar monastic manuscript, containing extracts from the writings of Cassiodorus, and other primitive fathers of the church.

"In this collection there is a prayer to St. Nicaise, which seems to have been intended for the consecration of amulets made by nuns, and inscribed with his name, to be worn as a protection against the Small Pox. It should be allowed, in charity to our forefathers, that such an ecclesiastical composition was of a very ancient date. This copy was probably written in the tenth century; as it is followed by a calendar of the paschal terms, beginning with the year 988, and continued by successive hands to the year 1268. It is in barbarous Latin, with a chorus of unmeaning syllables for chanting, as was the practice of the monks, and may be rendered into English, as follows:—

"In the name of our Lord Jesus Christ, may the Lord protect these persons, and may the work of these virgins ward off the Small Pox. Saint Nicaise had the Small Pox, and he asked the Lord (to preserve) whoever carried his name inscribed.

"'O, Saint Nicaise! thou illustrious bishop and martyr, pray for me a sinner, and defend me by thy intercession from this disease. Amen.'

"As it is asserted in this prayer, that Saint Nicaise, who had been bishop of Rheims in the fifth century, had the Small Pox, it was important to investigate the fact. In the Lives of the Saints by Surius, there are two of Saint Nicaise. One is very ancient, but anonymous; the other is the most copious, and is extracted from the works of Flodoard, who was born at Rheims, and wrote a full History of the Church of that City.

"He relates, that when an army of Vandals had entered Rheims and were massacring the inhabitants, the benevolent bishop, arrayed in his ecclesiastic robes, and accompanied by Eutropia, his virgin sister, devoted themselves to stop the fury of these infidels,

---

in adjutorium sit Salvator noster *N°* dominus celi..... audi preces famulorum famularumque tuarum Domine Jhesu Chrispte.... adque peto Angelorum milia aut (ut) me *N°* salvent ac defendant doloris igniculo et potestate Variola, ac protegat mortis a periculo; tuas Jhesu Chrispte aures tuas nobis inclina.' &c. Bib. Harleian, lib. MSS. num. 585, p. 202.

 and and to save the lives of the citizens. But the ruffians were neither overawed by the venerable bishop, nor melted by the beauty of the maid: for, while he was exhorting them to spare the people, they transfixed him with their spears, and laid him dead at his sister's feet.

"At this spectacle, Eutropia raised her imploring eyes to heaven, sunk on her knee, exposed her naked neck to the swords of the Vandals, supplicating that they would only kill her. She obtained some mercy, for one of the least inhuman of these barbarians cut off her head, in sport.

"These murders were, in the language of that age, termed martyrdoms, and the bishop and his sister were canonized. Saint Nicaise was deservedly regarded as the glory of the church and city of Rheims, and Flodoard has collected every particular of his life that was known: but not a syllable is mentioned of his having had the Small Pox. That assertion in the Anglo-Saxon prayer can therefore only be considered as one of those pious frauds which were so frequent in the dark ages. A saint was wanted to superintend this new disease, and Saint Nicaise was accidentally pitched upon by the ignorant Monks; who, to justify their choice, asserted that he had a disease which did not appear in Europe till about three centuries after his death."

---

*On the uncertain Date of Small Pox and some other Diseases, with a Digression.*—"In the eleventh century, Constantinus Africanus in Italy, and Avenzoar in Spain, published their works, in which are included discourses on Small Pox, as an ordinary malady. Notwithstanding which Dr. Mead, Baron Dimsdale, and many others, have maintained that the Small Pox was brought into Europe by the Crusaders, who did not set out on their frantic expedition until the year 1096.

"This opinion had no foundation, either in reasoning or in history. For, although the contagion of Small Pox might be very readily carried by an invasion, to the most remote countries: yet it is not likely to retrograde upon the country of the invaders, by means of the returning survivors: because the contagion acquired abroad would be dispersed before they could reach their homes, as was formerly noticed. The Small Pox in fact reached Europe more than two centuries before the Crusades; and the historians of the holy wars take no notice of the Christian armies having suffered from that malady. In searching them, only a single trace of it was detected in the following description by Bernard, of the person of the Count Joscelyn, grandson of Atho, the founder of the illustrious family of Courtney. "This count was small in stature, his limbs were finely formed, his hair was brown, and his countenance pleasing, *though pitted with marks of the Small Pox;* his eyes were large, and his nose aqueline. He was gallant and fierce in battle, but loved the pleasures of the table, and was too luxurious." These Small Pox marks might have been been acquired in early life in France, or the historian probably would have omitted that defect in describing so favourite a knight.

"His death occurred in 1152, and was so memorable as to justify a digression.

"When Fulco reigned at Jerusalem, Joscelin, Count of Riez, was occupied in the siege of a Turkish town; and, having undermined the wall, it suddenly fell down and involved him in the ruins. The soldiers seeing the Count in danger, ran to his assistance, removed the stones and earth with which he was oppressed, and carried him to his tent on his shield.

"But the physicians soon perceived that the vital parts were irrecoverably injured: and, while he lay declining and languid, accounts were brought that the soldan of Cumania had dared to lay siege to Cherson, a town under the Count's jurisdiction. Indignant at this insult, yet incapable of taking the field, he sent for his son; commanded him to assemble the army, and to march instantly to the relief of Cherson. His son remonstrated against the measure; and urged, that their forces were too few to encounter so numerous an host of Turks. The father was deeply mortified to find that a youth begotten by him, and the heir of his Earldom, should possess a pusillanimous soul. Then, rousing himself, he gave orders to collect the troops; and, as soon as they were drawn up in array, he was lifted into a car, and proceeded at their head against the enemy.

"Before he came in sight of Cherson, some returning scouts brought intelligence, that the soldan, having heard of his advance, had suddenly raised the siege and retired. Upon this the count halted the army, and, all his remaining powers which he had so strenuously exerted now failing him, he raised his trembling hands to heaven, and prayed thus: 'O! most clement Father, I thank thee for having exalted me to high honours, and especially for this last; that even when thus changed and sinking into the grave, my enemies have fled at my approach, and have abandoned my province. I know and acknowledge, O most gracious God! that these are thy works alone!' Having pronounced these words, he sunk down in his car in the presence of the army, and died.

"This was a Paladin.

"It was formerly noticed, that there is no trace of Small Pox to be found in the lives of the early saints, because that malady had not then reached Europe. But even after it had spread through this quarter of the globe, the saints appear to have been peculiarly negligent of their Small Pox patients. However, in the year 1218, there lived in France a distinguished female, who was entitled Saint Franca, and who wrought abundance of miracles. Among the rest, she restored sight to a person who had been rendered blind by the Small Pox. And Saint Ivo, also, about the year 1303, miracuously cleared off a spot from the eye of a young girl, which had been caused by a variolous pustule. It is likewise recorded, recorded, that Pope Urban V. cured a patient affected with the fever of Small Pox, about the year 1364.

"This being considered a miracle, is a proof of the fatality of the disease at that time. Though in the fifteenth century miracles were declining fast, yet there was a woman in France, who had lost the sight of both her eyes from the Small Pox, that had the good fortune to stumble upon Saint Jacob Philip, who restored her vision to perfection: and another, who had been blind for three years from the same cause, was cured by Saint Cunera. The Lives of the Saints, compiled by Bishop Surius, and the Bollandini, which contain those facts, are now held in little reverence. But, independently of medical authorities to be noticed afterwards, these scattered passages are the only very early allusions to Small Pox, which, after a strict search, have been found out; for no formal information upon this subject has been given by cotemporary historians.

"In the fifteenth century a greater attention began to be paid to the discrimination of diseases: for Mezeray states, in 1414, the commencement of a malady of far less importance than the Small Pox. His words are, 'That a strange kind of rheum*, named the Hooping-cough, tormented all sorts of people during the months of February and March, and rendered their voices so hoarse, that the bar, the pulpits, and the colleges were mute. All the old men who were seized with it died.'

Notwithstanding this learned remark, and ready as we are to admit that the term *coqueluche* is the present vernacular name for Hooping-cough, yet we strongly suspect that the disease referred to by Mezeray was an epidemic catarrh or influenza. First, because no mention is made of *toux*, but only *rhûme*. 2dly, because the disease seems to have continued epidemic for only two months at most. 3dly, because it induced so sudden and universal a hoarseness as to silence the bar and the pulpit and the collegiate chairs. Lastly, because it is described as universally fatal to all the *old people* who were attacked, without any mention of children. We say nothing concerning the general use of the term

---

* "'Un estrange rhûme qu'on nomme La Coquelûche tourmenta toutes sortes de personnes durant les mois de Fevrier et de Mars et leur rendit la voix si enroué, que le Barreau, les Chaires et les Colleges en furent muets. Il causa la mort à toutes les Vieillards qui en furent attents.' Abreg. Chronol. de l'Hist. de France, par Mezeray; tom. ii. p. 651.

"The vernacular words of Coqueluche, Hooping-cough, and Kin-cough, are more expressive and correct, than the learned names, Pertussis or Tussis convulsiva. Nosology and chemistry have been obscured by a multitude of varying scientific nomenclatures.

*coquelûche*

*coquelúche* for any epidemic disease, because that may be a modern figure.

We perfectly agree, however, with the learned author, in his note below, that nosology has hitherto only added to the obscurity of our art, which did not need any thing to increase its difficulties.—EDIT.

---

### Mr. JOHN LAURENCE's *improved Pair of Crutches for Lame Persons.*

*Fig. 1.*

*Fig. 2.*

*Fig. 3.*

Fig. 1 represents the whole crutch, with it moveable handle *a*, in which the peculiar merit of this invention consists; this is shewn on a larger scale at fig. 2, where the handle with its screw is seen, by which it can be fixed at any height or position required by the patient, who is thus enabled to hold the crutch with far greater ease than in the usual manner of constructing it without such handles.

Fig. 3 shews the manner in which the head of the crutch is formed, so as to produce an elastic support to the patient, and thus break off the effects of the shocks occasioned by striking the end of the crutch upon the ground in walking with it; this is done by making the wood in the form of a crescent, across which, bands of linen girth web are stretched and secured firmly at each end, and the vacancy underneath them being with a cushion of twisted and baked horsehair: the whole, when covered with plush, velvet, &c. nailed over it, forms a very pleasant support to the patient: the lower end of the crutch has a turned conical piece of wood affixed to it, which may be renewed when worn away, or lengthened to suit the height of the patient.—*Soc. of Arts.*

CRITICAL

## Figures and Tables

**Figure f1:**
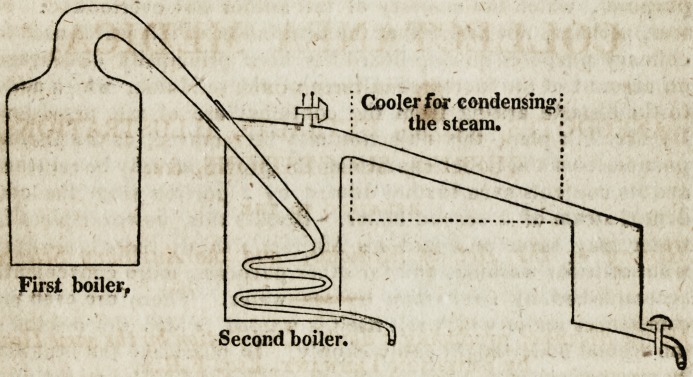


**Fig. 1. Fig. 2. f2:**
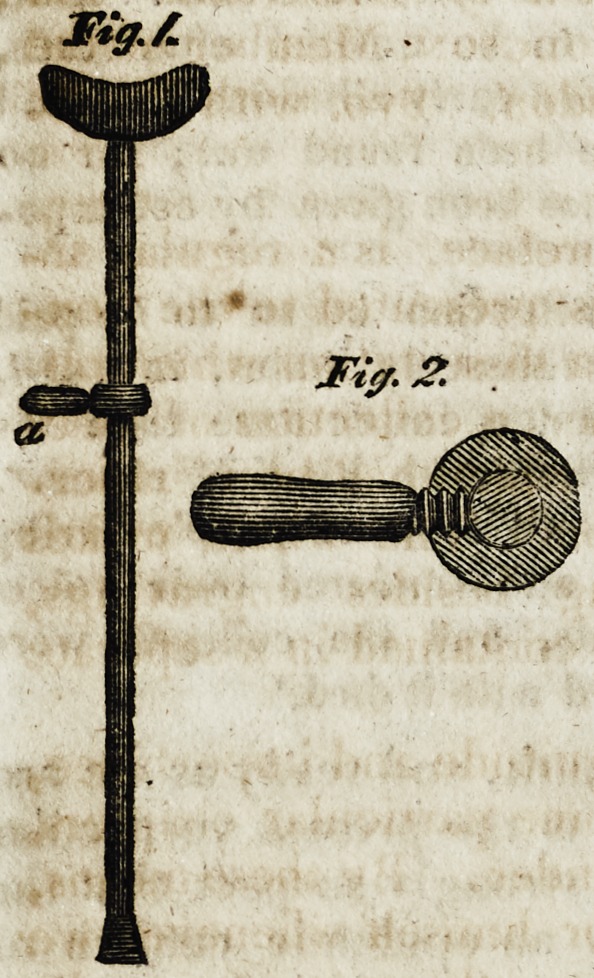


**Fig. 3. f3:**